# Effectiveness and Safety of Tasso+ Pediatric Microsampling for Child Blood Lead Levels: Cohort Study

**DOI:** 10.2196/101700

**Published:** 2026-10-22

**Authors:** Alana M W LeBron, Kristina Uban, Julia Mangione, Olaya Daki, Erika Ramirez-Mayoral, Maya Cheav, Sarahi Gutierrez Luna, Adriana Orellana, Yohana Rojas de Jesus, Yezenia Marrujo, Blanca I Contreras, Teresa Campos, Jun Wu

**Affiliations:** 1 Department of Health, Society, and Behavior, Joe C. Wen School of Population & Public Health, University of California, Irvine, 856 Health Sciences Quad, Office 3555, Irvine, CA, 92697, United States, 1 949-824-5242, 1 949-824-1019; 2 Department of Chicano/Latino Studies, School of Social Sciences, University of California, Irvine, Irvine, CA, United States; 3 Institute of Interdisciplinary Salivary Bioscience Research, University of California, Irvine, Irvine, CA, United States; 4 Center for the Neurobiology of Learning and Memory (CNLM), University of California, Irvine, Irvine, CA, United States; 5 Community Member At-Large, Santa Ana, CA, United States; 6 Orange County Environmental Justice, Santa Ana, CA, United States; 7 Department of Environmental and Occupational Health, Joe C. Wen School of Population & Public Health, University of California, Irvine, Irvine, CA, United States

**Keywords:** biosampling, pediatric research, Tasso, clinical research, environmental health, methodology, epidemiology, adverse events, child blood lead levels

## Abstract

**Background:**

The growing interest in promoting child health requires minimally invasive biospecimen collection. Particularly during and since the COVID-19 pandemic, there has been increased interest in microsampling technology to democratize participation in research involving biospecimens, particularly for children. These advances mitigate barriers, such as scheduling, transportation, and psychosocial challenges. Tasso is a microsampling biomedical device that has gained popularity in research. It uses a vacuum-assisted capillary system that attaches to the upper arm and is considered minimally invasive, as it requires no venipuncture or phlebotomist. Yet, important gaps remain in understanding Tasso performance, particularly among pediatric populations where its administration is limited.

**Objective:**

This research addresses a significant gap in biomedical device development by examining the effectiveness, safety, and failure rate of the Tasso+ Class II medical device designed for blood sampling (puncture) among children. The Tasso+ was selected for a pediatric cohort research study given advertisement of methodological advantages over traditional phlebotomy. This original investigation characterizes the effectiveness of and potential for institutional review board–designated adverse events, following the use of the Tasso+ Class II medical device within a pediatric population.

**Methods:**

Blood collection was essential to measure lead (Pb) exposures for children residing in a region known to be polluted. Parents/guardians consented, and children assented to Tasso+ sampling on the upper arm. The Tasso+ device was administered in 2024 by trained researchers on 2 pilot participants (ages 7‐8 years) and subsequently 13 study participants (ages 7‐10 years). Sampling was terminated upon participant reports of persistent pain and scarring on children’s arms. Follow-up was conducted at intervals after device application: 2‐11 weeks and 10‐19 months. Data sources included data collection and laboratory records and survey-based follow-up with participating families. Analyses involved descriptive statistics drawn from data collection and laboratory records and descriptive statistics and content analysis of a follow-up survey administered to participating families. Content analysis of open-ended responses to the follow-up survey administered to families identified themes and their frequency of occurrence. Main measures included the device failure rate, effectiveness (ie, blood volume), and adverse outcomes at the arm puncture site.

**Results:**

The device failure rate was 26.7% (4/15), with only 66.7% (10/15) and 20% (3/20) of the cases having the optimal (>450 µL) or adequate (250‐449 µL) blood volume, respectively. At initial follow-up, 23.1% reported ongoing pain; none reported ongoing pain at extended follow-up. Scarring showed high initial incidence (61.5%) and persistence (75.0%). Children reported shame related to the participant-reported and researcher-observed scar and impacts of pain on feelings toward physical touch (eg, hug/embrace).

**Conclusions:**

These effectiveness barriers and adverse events highlight the need for documentation to inform the future rollout of novel devices for participatory research, longer-term follow-up, and addressing potential gaps in transparency between manufacturers and researchers who use biomedical devices.

## Introduction

Growing interest in promoting child health requires minimally invasive biospecimen collection. Blood samples are the gold-standard biospecimen in biomedical research, with venipuncture being the most common and reputable approach. In pediatric research, barriers to venous blood draws include fear of needles, pain, inadequate blood volume, emotional burden, collecting within a standard clinic/laboratory, and pediatric phlebotomy expertise needed given children’s small and less predictable veins [1-5].

Particularly during and since the COVID-19 pandemic, there has been growing interest in microsampling technology to democratize participation in research involving biospecimens, particularly for children [6-8]. These advances mitigate barriers, such as scheduling, transportation, and psychosocial challenges. Interest stems from the goal of making biospecimen collection more accessible so that research participants or trained nonphlebotomist researchers can collect biospecimens [9,10]. Another appeal is the potential to reduce pain and to prevent or minimize adverse events from biospecimen collection [11].

Microsampling biomedical devices, such as the Tasso+, have grown in popularity given the potential to overcome these barriers [10,12,13]. The Tasso uses a vacuum-assisted capillary system that attaches to the upper arm and is considered minimally invasive as it requires no venipuncture needle or phlebotomist and instead uses a puncture-based needle [14,15]. These design advancements address concerns such as venipuncture-related pain and adequate blood volume [14], enable self-administration, address transportation and scheduling challenges, and eliminate the need for a phlebotomist [6,7,9,10,15-17].

Yet, important gaps remain in understanding Tasso performance, particularly among pediatric populations where Tasso device administration is limited [6,8,9,17]. In a study of Tasso’s feasibility, acceptability, and safety conducted by Dasari et al [6], the sample included adults and children (1‐17 years old). Parent-child dyads in this study showed adequate capillary blood volumes and low-to-moderate pain for children (parent reported). No adverse events were reported (eg, bruises, itchiness, swelling, tenderness) among children. Furthermore, the device failure rate (ie, collection of <100 μL, device malfunction, coagulation issues) was only 4.4% for the entire sample (children and adults). While other studies have used Tasso with children [6,9,17], to our knowledge, no published studies have assessed long-term adverse events after Tasso administration or the occurrence of scarring. Previous assessments monitored adverse events 24 hours post-collection [6].

While advancements in vacuum-assisted capillary microsampling devices hold promise, what remains unknown is any delayed or persistent effects following Tasso use beyond 24 hours. Capturing longer-term outcomes (eg, beyond 24 hours) following biomedical device use requires significant resources and established relationships with research participants. Community-based participatory research (CBPR) approaches are rooted in building long-term relationships that engage communities and research participants as active (rather than passive) research partners [18]. CBPR approaches are uniquely positioned to assess longer-term outcomes of microsampling device use. At the time of the present study, Tasso+ was cleared by the US Food and Drug Administration for research purposes with adults and investigational use with children [12,19]. The Tasso protocol indicated the device could be used in pediatric research [8,19], and potential risks include the possibility that “minor bruising or residual marks may occur at the sample collection site” [20].

The present study’s primary objective was to assess the Tasso+ device failure rate, effectiveness, and adverse events associated with Tasso+ use as part of an ongoing prospective cohort study of children’s lead (Pb) exposures. Specifically, this nested study (adverse event study within the larger prospective cohort study) focuses on Tasso+ device use and outcomes with 15 participating children before the use of the Tasso+ device was halted. We examined the frequency, severity, and nature of unexpected adverse events, based on follow-up 2‐11 weeks and 10‐19 months after the administration of the Tasso+. The findings have implications for child health and safety, and for the broader framework of how biomedical devices are tested, approved, and marketed for pediatric populations.

## Methods

### Study Design

This adverse event study is a study within an ongoing prospective observational study, Inequities in Child Lead Exposures and Academic and Neurobehavioral Outcomes (I-CLEAN), which uses a CBPR approach to examine child lead exposures and academic and neurobehavioral outcomes. I-CLEAN partners include Orange County Environmental Justice, Getting Residents Engaged in Empowering Neighborhoods-Madison Park Neighborhood Association (GREEN-MPNA), and public health scholars. Guided by CBPR principles [21], community and academic partners share decision-making throughout the research process (eg, research questions, study design, data collection, analysis, interpretation, dissemination) [22,23]. From November 2023 to March 2024, we held community-academic planning and capacity-building sessions to discuss and strategize research ethics considerations; community engagement; consent/assent; and data collection protocols, including Tasso+ administration following the manufacturer’s guidelines.

Research staff were trained to administer the Tasso+ first, piloted first on adult researchers, and then on pilot child participants from December 2023 to February 2024. Tasso+ was administered to research study participants in May to June 2024, with extended follow-up through September 2025.

Drawing on the earlier phase of the I-CLEAN prospective observational study that temporarily used the Tasso+ Class II medical device to assess children’s lead exposures (December 2023-June 2024), this original investigation characterizes device failure rate, device effectiveness, and institutional review board (IRB)-designated adverse events within a pediatric population. Device use was halted after a sample size of 15 (including 2 pilot participants and 13 participants from the prospective cohort study). We followed the STROBE Statement-Checklist for cohort studies (Checklist 1).

### Study Population

The longitudinal I-CLEAN study is actively recruiting children, with an enrollment goal of 600 families. Initial inclusion criteria (which had since expanded) included that children be 7‐10 years of age; reside within a Santa Ana, CA ZIP code, which has documented soil lead contamination [23]; attend a Santa Ana Unified School District school; have one parent/guardian who is fluent in English, Spanish, or Vietnamese; and have the parent/guardian consent and child assent for child blood testing and the donation of a naturally shed baby tooth. Participants were actively recruited through in-person interpersonal outreach within the research partners’ networks and through community-based recruitment (eg, elementary schools, community events). The initial phase involved administering the Tasso+ to 15 children (ages 7‐10; 2 were pilot participants who were members of the researcher’s networks). Several adverse events contributed to the need to halt Tasso+ use beyond these 15 individuals.

### Exposure: Tasso+ Device

Tasso+ was selected for its minimally invasive, capillary puncture–based needle (vs venipuncture needle), compact, lightweight, and user-friendly design, potentially reducing psychological and logistical barriers associated with venipuncture. Tasso+ involves a single-use sterile blood lancet that leverages sterile adhesive to attach to the upper arm. The button-activated lancet creates a shallow puncture and leverages vacuum-assisted technology to collect capillary whole blood into the collection reservoir within 5‐10 minutes. Furthermore, Tasso+ enables the use of various microtubes (eg, EDTA, serum separator). These factors make Tasso+ a tool for use in nonclinical (eg, home, community-based) settings for quick blood draws that do not require specialized phlebotomists. In adults, Tasso is advertised as a self-sampling device. Given the pediatric population, all child sampling was administered by trained research staff.

Training involved (1) implementing manufacturer recommendations to inform internal training, (2) designing protocols in conjunction with Tasso representatives, (3) piloting the protocol with 2 pediatric volunteers (administered by trained staff) and 2 adult researchers (1 self-sampling, 1 administered by trained researcher), (4) refining the protocol based on the pilot and further consultation with Tasso representatives, and (5) conducting 2 team trainings (1.5-hours each) to practice sampling on adult researchers.

### Data Collection

The Tasso+ was administered by trained researchers in a private room of a community center or participant’s homes. Within 1 week after Tasso+ collection, researchers called participating families to address any questions about the study and to check on the participating child after blood sampling. Adverse events associated with the Tasso+ came to the study’s attention when 2 participating promotoras (Community Health Workers) shared on July 15, 2024 (7 and 10 weeks, respectively, after blood sampling), that their children had parent-reported scars at the location where the Tasso+ was administered. Additionally, a participating parent informed the research team on July 20, 2024 (4 weeks after blood sampling) of ongoing pain and parent-reported scarring at the location where the Tasso+ was administered. The unanticipated problem was formally reported to the IRB on July 23, 2024. The IRB designated these unanticipated problems as adverse events. In the present study, scarring refers to participants’ and trained researchers’ descriptions of skin markings on children’s arms.

This study draws upon data from a longitudinal Tasso+ adverse event assessment, as well as existing study records regarding data collection visit documentation and lab records pertaining to blood samples. The adverse event initial follow-up assessment was conducted from July 2024 to August 2024, from the date of the notification of the first adverse event until we called participants up to 3 times (median: 53, IQR : 32-53 days from administration of Tasso+ device). Research staff conducted initial follow-up assessments with families by phone (n=11) and in-person (n=2) 2‐11 weeks after Tasso+ administration to assess parent-reported pain, discomfort, or scarring.

The adverse event extended follow-up assessment was conducted from April 2025 to September 2025, 10‐19 months post-collection, beginning approximately 3 weeks after IRB approval (to ensure adequate protocol training and based on participant availability) until we exhausted outreach to each family (median: 347, IQR : 322-396 days from administration of Tasso+ device). Among the 15 participants who completed blood collection with the Tasso+ between December 2023 and June 2024, we completed in-person (eg, community center, participant’s home) extended follow-up assessments with 11 of the 15 participants and conducted a remote assessment with 1 participant. (Below, results are reported out of 12 total successfully conducted extended follow-up assessments.) One participant moved out of the study area, and 2 participants were lost to follow-up despite multiple contact attempts (phone calls, texts, home visits).

*Failure rate* is derived from data collection visit documentation of whether the Tasso+ device was able to draw blood and the number of attempts, which included up to 2 attempts to administer the Tasso+, up to 1 administration per arm.

*Blood volume*, a measure of device effectiveness, was assessed by the volume reported on the microcontainer and was documented in the lab by a trained researcher. To verify the blood volume within 6 hours of sample collection, after taring the scale with an unused microtainer, the sample collection container was weighed in the lab. (We recognize volume and weight are not perfect one-to-one conversions.)

*Adverse events*: The adverse event initial follow-up assessment included questions about how the child’s arm felt after the visit (open-ended response, with a probe about any experiences of pain), duration (open-ended response), and marks or scarring (yes/no response options, with the opportunity for participants to elaborate).

For the extended adverse event follow-up assessment, with permission from the parent/guardian and child, trained researchers physically inspected the arm at the location where the Tasso+ was administered and conducted an adverse event extended follow-up survey with the child and parent/guardian. Closed-ended survey questions assessed potential experiences with pain, scarring, physical differences, range of motion, and seeking medical attention. The extended follow-up survey also captured responses to open-ended questions about any potential side effects of use of the Tasso+. Specifically, participants were asked about any other symptoms or issues that they would like to report. Additionally, trained researchers took a deidentified photo of the respective arm, with permission from the child and parent/guardian.

### Data Analysis

Descriptive statistics included frequencies/percents (categorical) and means/SDs (continuous). While the ongoing prospective observational study currently includes 251 participants, the sample size for this adverse event study is 15 due to termination of Tasso+ use after the adverse events. The small sample size precluded formal statistical significance testing.

Content analysis was conducted on extended follow-up survey responses to open-ended questions about any potential side effects of use of the Tasso+. The dataset for this analysis includes 17 out of 24 possible extended follow-up responses—the complete set of responses available for analysis. This includes responses at extended follow-up from children (9 open-ended responses out of 12 possible responses that elaborated on physical symptoms/issues) and parents/guardians (8 out of 12 possible responses, with 8 out of 8 responses elaborating on physical symptoms/issues) [24]. Given the novelty of the adverse event and exploratory nature of the content analysis (17 responses total), 2 coders (AMWL, JM) conducted inductive (data-driven) coding of open-ended responses to the extended follow-up survey regarding parent/guardian reports of any potential side effects of use of the Tasso+ device [25]. Following 2 reviews of the open-ended survey responses, the lead coder (AMWL) developed the codebook, which involved inductive codes. Both coders (AMWL, JM) applied the codebook to the open-ended responses to the survey. Inductive codes included “characterizations of pain or discomfort,” “characterizations of scarring/physical descriptions,” “characterizations of itching,” “duration of symptoms,” and “indication of no symptoms.” The coders then organized the codes into broader categories and themes, including physical side effects of the adverse event and psychosocial side effects of the adverse event. The themes and their frequency of occurrence were then evaluated.

Steps to strengthen the credibility and trustworthiness [26] of the findings included (1) the coding team discussed the emerging qualitative findings with the larger study team, including academic scholars, staff at partner community–based organizations, and promotoras affiliated with the study; (2) participant checks with parents participating in the study; (3) debriefing with research staff involved in the adverse event interactions with study participants; and (4) revisiting the coding scheme and open-ended responses to validate the application of the inductive codes.

### Ethical Considerations

The prospective observational study and adverse event follow-up assessment received University of California, Irvine IRB approval (Protocol #2385). Adult parents/guardians provided written informed consent, and children provided verbal assent to the prospective observational study, which detailed risks and protections pertaining to participant privacy and confidentiality. The IRB did not require consent for the adverse event assessment. All participants received cash incentives for completion of surveys at the baseline visit (US $40) and provision of a blood sample (US $45) as an expression of appreciation for their time and their contributions to the study. Data are deidentified. Paper copies are stored under 3 layers of locking. Electronic data are stored on a secure server.

## Results

### Participant Demographics

Approximately half of participating children were male. Participants were predominantly Latino and from low- to moderate-income households (Table 1).

**Table 1.** Participant demographics, I-CLEAN (Inequities in Child Lead Exposures and Academic and Neurobehavioral Outcomes) study, 2024 to 2025 (N=15).

Characteristic		Values
Child age (y), mean (SD; range)		8.9 (0.7164222; 7.9‐10.3)
Child race/ethnicity, n (%)		
Latino		13 (87)
White, non-Latino		2 (13)
Child sex assigned at birth, n (%)		
Female		7 (47)
Male		8 (53)
Household income (US $), n (%)		
<10,000		3 (20)
10,000-20,000		0 (0)
21,000-40,000		3 (20)
41,000-60,000		7 (47)
>61,000		2 (13)

### Device Failure and Effectiveness

We experienced device failure with approximately one-quarter of participants, substantially higher than the manufacturer’s reported failure rate of <5% (Table 2). The need for second attempts on the opposite arm for some participants after device failure increased costs (US $25/additional device). Notably, each of the 3 children who needed a second attempt also experienced an adverse event. One participant declined a second attempt at administering the Tasso+ device, leading to a complete blood collection failure.

Blood volumes ranged from 0 to 700 µL, with a mean of 420 (SD 167) µL. However, one-quarter of successful collections yielded suboptimal blood volumes, compromising analytical sensitivity for trace metal detection. Weight-based measurements confirmed these findings, accounting for preloaded anticoagulant in tube volume markings.

**Table 2.** Tasso+ blood collection failure rate and effectiveness in 15 pediatric participants, 2023 to 2024.

Outcome measure	Participants, n (%)	Details
Consent and participation		
Consented to Tasso+	15 (100)	All participants initially agreed
Participants in Tasso+ follow-up		
Initial follow-up	13 (87)	—^a^
Extended follow-up	12 (80)	—
Collection outcomes		
Successful on the first attempt	11 (73)	—
Failure on the first attempt	4 (27)	—
Involved a second attempt	3 (20)	A total of 4 participants were eligible for a second attempt due to device failure on the first attempt. One of the 4 participants declined a second attempt. The remaining 3 participants had successful blood collection on the second attempt.
Complete collection failure	1 (7)	Did not attempt second collection
Blood volume obtained (µL)		Median (IQR) weight: 0.488 (0.445-0.535) g
≥450 (optimal)	10 (67)	Actual weight range^b^: 0.456‐0.743 g
250‐449 (adequate)	3 (20)	Actual weight range^b^: 0.384‐0.445 g
1‐249 (inadequate)	1 (7)	Actual weight value^b^: 0.146 g
0 (failure)	1 (7)	Collection failed
Technical performance		
Samples meeting minimum for analysis	12/13^c^ (92)	≥250 µL required
Required multiple devices per child	3/14^d^ (21)	Additional cost and trauma

a Not applicable.

b Since initial blood volume from tube markings included preloaded anticoagulant, tubes were weighed (grams) on an Ohaus Scout compact bench scale for a more accurate assessment of blood volume, with blood density (~1.06 g/mL or mg/µL) making weight approximately equivalent to volume.

c As the first 2 pilot participants’ samples were not included in analysis, the denominator here represents the total number of samples analyzed after collection.

d The denominator excludes the participant who did not consent to a second attempt on the other arm.

### Physical Impacts

Shown in Figure 1 are the longitudinal patterns of the adverse events at (1) initial follow-up assessment, 2 to 11 weeks after Tasso+ administration, and (2) extended follow-up assessment, 10 to 19 months post collection. Nearly one-quarter of participants who participated in initial follow-up reported pain initially following Tasso+ use; however, no participants reported ongoing pain at the extended follow-up assessment. Scarring showed both high initial incidence and persistence. Over half of parents/guardians reported scarring at the initial follow-up assessment. At extended follow-up, all cases of initial parent/guardian-reported scarring still presented with visible scarring (as assessed by a trained researcher), and a trained researcher identified scarring in 1 additional participant at in-person follow-up, indicating that the majority of extended follow-up participants had persistent parent/guardian-reported or researcher-observed scarring (see Figure 2 for examples of reported scarring). The persistence of parent/guardian-reported and researcher-observed scarring exceeds typical wound healing timelines [27], suggesting that parent-reported and researcher-observed scarring from the Tasso+ represents a longer-term adverse event rather than a transient healing response. At initial follow-up, more than one-third of the participants reported a bump (see Figure 2 for examples of bumps), and nearly one-third reported itching. At extended follow-up (in-person), additional adverse events emerged: half had researcher-observed bumps, 2 reported discomfort, and 1 reported itching. Additionally, 2 families reported redness or swelling for weeks or months after Tasso+ use. The data underscore that while long-term pain was rare, the development of lasting physical marks on children’s skin (as reported by parents/guardians and observed by trained researchers) represents the primary safety concern with this device in pediatric populations.

**Figure 1.**
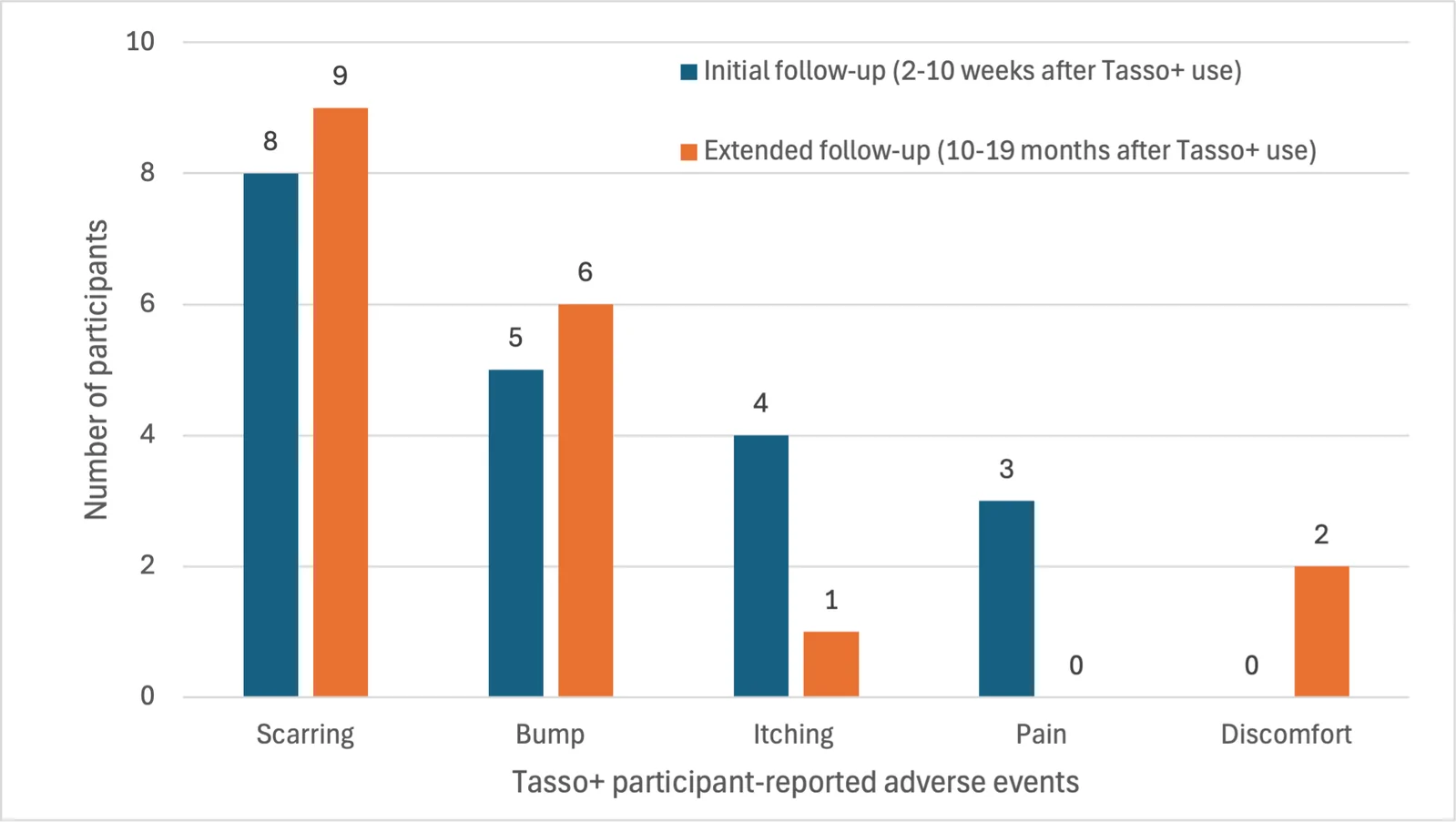
Count of adverse events in a pediatric population (7-10 years of age) after administering the Tasso+ device for blood sampling, measured 2 to 11 weeks and 10 to 19 months after administration, 2024 to 2025. Initial follow-up data (2‐11 weeks after Tasso+ administration) involve 13 participants; extended follow-up data (10‐19 months after Tasso+ administration) involve 12 participants, with in-person follow-up completed for 11 families and remote follow-up completed for 1 family. The increase in the number of scarring cases is due to physical inspection of the arm by a trained researcher at the extended follow-up assessment, compared to parent/guardian self-report by phone at the initial follow-up assessment. The increase in some adverse outcomes (eg, bumps, discomfort) is linked with the addition of survey items to the extended follow-up survey, informed by research conversations about more latent adverse outcomes that were not included in the initial follow-up survey’s closed-ended questions.

A total of 16 out of 17 open-ended responses included participants’ elaborations on negative symptoms or issues that participants linked with administration of the Tasso+ device. Counts reflect responses rather than unique participants; a child and parent/guardian from the same family may describe the same event. The most frequent open-ended responses pertained to long-term parent/guardian-reported scarring (11/17 responses), with participants characterizing the scar as a *“*little scar*”* (parent participant A) and “It’s (scar) the same […] it hasn’t changed” (parent participant B). The second most frequent response pertained to mentions of pain (4/17 responses). When discussing pain at the arm or arms where the Tasso+ was administered, 2 parents/guardians reported children being sensitive to touch for several weeks. For example, parent participant C reported, “[he] said his arm hurt when [he] touched in the spot of the blood sampling for about 2‐3 weeks after [administration of the Tasso+ device].” Parent participant D reported, *“*[the] pain lasted months, [it] came and went.” Discussions of itching (3/17 responses) were the third most common parent/guardian report. For example, parent participant D recalled that itching followed the pain, noting “Then (after the pain) itching, [and] the child scratched.” In addition to these physical harms of Tasso+ use with children, in open-ended responses to the follow-up survey, 1 exploratory finding was that participants reported several psychosocial harms related to reported physical pain, discomfort, and/or scarring after Tasso+ administration. In one case, child participant D was in so much pain that they did not want to receive a hug from their family members. This participant also reported experiencing so much shame about the scar that they did not want to wear sleeveless tops and reported fear of the research team.

**Figure 2.**
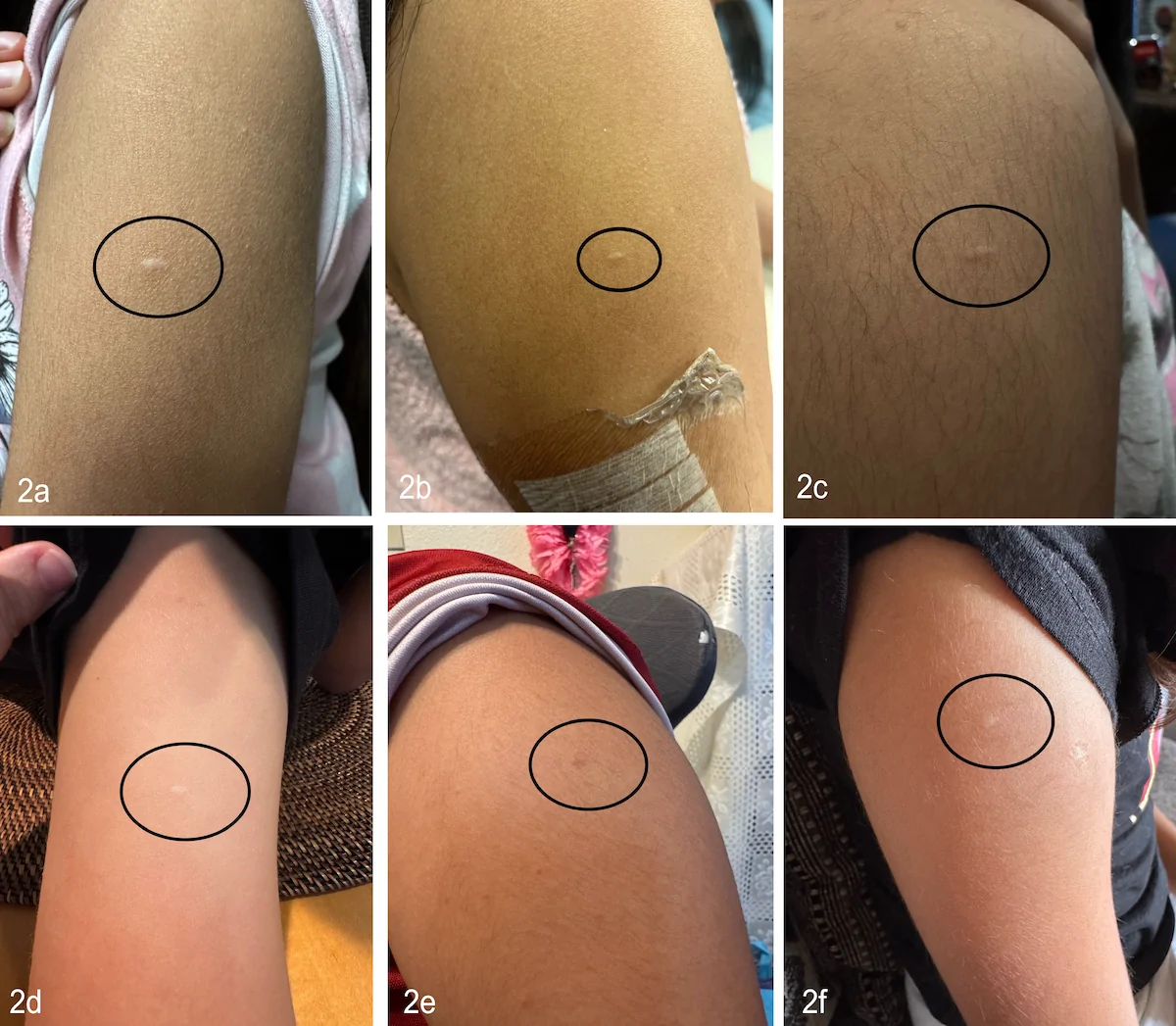
Images of scars and raised or hardened bumps from administration of the Tasso+ device for capillary blood sampling in pediatric population 7 to 10 years of age, 2024 to 2025. (A-C) Pediatric study participants with a raised or hardened bump and scar at the location on the child’s arm where the Tasso+ blood sampling device was administered. (D-F) Pediatric study participants with a scar at the location on the child’s arm where the Tasso+ blood sampling device was administered. Parents/guardians and children provided permission for trained research staff to take a deidentified photo for documentation and publication.

## Discussion

### Principal Findings

This study documents longer-term outcomes of Tasso+ use for capillary blood sampling with pediatric populations, including the failure rate, effectiveness, and adverse events. Three key findings emerge. First, adverse events were demonstrated by prolonged pain and/or parent/guardian-reported and researcher-observed scarring at the puncture site of children’s arms where the Tasso+ was administered. Second, exploratory findings regarding psychosocial harms, such as shame about the reported scar, hiding the scar, and negative feelings toward physical touch (eg, hug), are lasting effects. Third, despite the manufacturer marketing Tasso+ as a low-cost device that can improve accessibility and blood sample collection, the 26.7% failure rate and 66.7% optimal blood volume collection suggest limited effectiveness.

### Comparison to Prior Work

Our findings contrast with the manufacturer’s reporting of device safety and the absence of literature detailing participant-reported or researcher-observed scarring from the Tasso+ for adult populations and limited reports of scarring for pediatric populations. The results highlight the importance of considering age-related differences in skin biology and wound healing for children in middle-childhood [28] and illuminate the importance of longer-term follow-up about adverse events associated with biomedical devices. The mechanisms of pain, scarring, and itching associated with use of the Tasso+ device with children remain unknown. Based on the findings from this exploratory study, we hypothesize that the puncture mechanism may play an important role. This hypothesis is informed by 3 key observations from this study. First, the relatively small size of the scars relative to the size of the Tasso+ device and location of the scars suggests that the experience of skin puncture and/or use of the larger metal component to puncture the skin may be involved in scarring. Second, several participants reported initial scabs, cuts, and itching at the location where the Tasso+ puncture mechanism was administered. Third, the vacuum suction and adhesive encompassed a much larger portion of the Tasso+ device, leading us to hypothesize that these device components may not play a major role in scarring. Additionally, the literature suggests that biological processes, such as inflammation and collagen production [28,29], may play a role in scarring. Future studies involving dermatologic evaluation are warranted that evaluate these post-hoc hypotheses of mechanisms by which Tasso+ device use may be connected with reported or observed scarring and that consider factors that may shape healing variation (eg, biological mechanisms, aftercare).

Tasso+ differs from other microsampling devices in two ways: (1) it uses a longer metal component to puncture the skin versus a standard small venipuncture needle point and (2) it uses vacuum suction to draw blood into the sampling vial. Whether the puncture method, vacuum suction, or both caused adverse events in this pediatric sample is unclear. We hypothesize that the high rate of adverse events associated with the Tasso+ in the present study may be due to the study’s longitudinal and CBPR nature that enabled capture of adverse events. We follow up with participating families after data collection events and maintain communication about research activities (eg, reporting results, scheduling year 2 visits). The present study is embedded in community-based settings at the leadership of community-based initiatives who have been central researchers throughout the study. Most studies that assessed adverse events associated with the Tasso+ did so immediately, or within 24 hours after administration [6,9], with none reporting systematic long-term follow-up. A study of 60 adult participants reported that 10% experienced minor healing issues, including small residual scars visible up to 3 months after self-collection [30]. Further, as Tasso+ is designed for self-sampling, other pediatric studies may have parents administer Tasso+. In contrast, this study trained researchers administered the Tasso+ device, potentially mitigating adverse event severity. Longitudinal systematic follow-up is warranted for future studies with adult and child participants to assess protracted adverse events (eg, scarring, pain, psychosocial, social, economic).

It is important not to essentialize or problematize race when interpreting scarring patterns for this pediatric population, which involves predominantly Latino children. Our findings spark hypotheses about the potential impact of Tasso+ use for children with developing epithelial cells and the lack of long-term follow-up to monitor adverse events. It is also important to understand how scars can be racialized. For example, community partners reported concern about discrimination in medical treatment and occupational opportunities for US-based immigrants who have visible tuberculosis vaccination scars received in their countries of origin. Substantiating these concerns, US immigrant populations are often framed in discourse and in policy as vectors of infections—patterns seen historically and with the pandemic [31-33]. While the present study documents longer-term (eg, 2‐11 weeks to 10‐19 months) outcomes from Tasso+ use, studies are warranted that examine wider consequences associated with scarring from the Tasso+.

### Implications

These findings have implications for child health and safety and for the broader framework of how biomedical devices are tested, approved, and marketed for use in pediatric populations. Biomedical device manufacturers should disclose the possibility of scarring up front. Additionally, manufacturers should provide aftercare instructions to prevent pain/scarring.

Our findings highlight the urgent need for enhanced, longitudinal, and CBPR assessments of adverse events associated with biomonitoring technologies. Tasso+ use increased during the pandemic, making adverse event dissemination urgent, particularly given that young children are not widely represented in peer-reviewed literature documenting Tasso+ outcomes. We aim to catalyze industry-wide collaboration (eg, with communities, dermatologists) toward developing more equitable and representative biomonitoring tools that effectively serve pediatric populations. This work contributes to the growing body of evidence supporting the imperative for participatory innovation in health care technology to improve health equity and patient outcomes.

### Limitations and Strengths

This study is characterized by some limitations. First, it analyzed experiences of a subset of participants from a larger prospective study of a pediatric population. The analytic size (n=15) is due to termination of Tasso+ use after adverse events were reported. These findings are based on participant reports and observations from trained researchers. Future studies are warranted that follow pediatric and adult populations longitudinally to understand long-term outcomes of biosampling biomedical devices and may benefit from collaborating with dermatology experts. Second, comprehensive follow-up required flexibility of the research team, including different modes (phone and in-person) and adapting survey items informed by emerging adverse events. Relatedly, the initial and follow-up assessment procedures differed. Accordingly, changes over time in reported or observed outcomes should be interpreted cautiously and understood as not directly comparable across time points. Future studies would benefit from prospectively and systematically tracking device failure, effectiveness, and adverse events, with regular longitudinal follow-up. Third, this sample lacks a comparison group (ie, venipuncture, other microsampling method), which would enhance assessments of whether these adverse events are unique to the Tasso+. Fourth, the open-ended responses were collected as part of adverse event safety extended follow-up that primarily focused on closed-ended responses/categories. The follow-up protocol was not designed as a larger qualitative study. While the qualitative methods provide important insights into psychosocial dimensions of the experience with the Tasso+ adverse events, given the small sample size, the findings should be understood as exploratory.

Some strengths are notable. First, the participatory approach, including collaboration with community leaders and community relationships, facilitated reporting adverse events. Given the troubled history of medical research with low-income and/or racially minoritized communities [34-36], relationship-based and participatory models of biomedical research help to overcome power imbalances between communities disproportionately burdened by health issues (eg, environmental exposures) and academic institutions. Second, the longitudinal nature of this study may have contributed to our ability to assess longer-term outcomes. Third, the manufacturer released a new product (Tasso Mini) as an improved version of the Tasso+, suggesting that the opportunity to document potential adverse events is narrowing and might continue to remain out of published literature.

### Conclusions

This is the first study to our knowledge that examines longer-term outcomes associated with Tasso+ use in pediatric populations. The findings indicate safety concerns and critical questions about the ethics of deploying inadequately tested medical devices in pediatric populations. Participatory and sustained research approaches are essential to ensure ethical, effective, and equitable implementation of biomedical technologies.

## Supplementary material

10.2196/101700 Checklist 1 STROBE checklist.

## Abbreviations

CBPR: community-based participatory research
GREEN-MPNA: Getting Residents Engaged in Empowering Neighborhoods-Madison Park Neighborhood Association
I-CLEAN: Inequities in Child Lead Exposures and Academic and Neurobehavioral Outcomes
IRB: institutional review board
STROBE: Strengthening the Reporting of Observational Studies in Epidemiology
